# Evaluation of Growth and Performance Traits in Limousin Weaned Bull Calves Using K-Means Clustering

**DOI:** 10.3390/ani16132075

**Published:** 2026-07-05

**Authors:** Márton János Demény, Dóra Lili Brassó, Natasa Fazekas, János Tőzsér

**Affiliations:** 1 Association of Hungarian Limousin and Blonde d’Aquitaine Breeders, Lőportár Street 16, 1134 Budapest, Hungary; demenymarton@gmail.com; 2 Albert Kázmér Faculty of Agricultural and Food Sciences of Széchenyi István University, Department of Animal Science, Vár Square 2, 9200 Mosonmagyaróvár, Hungary; tozser.janos@sze.hu; 3 Department of Animal Science, Institute of Animal Science, Biotechnology and Nature Conservation, Faculty of Agricultural and Food Sciences and Environmental Management, University of Debrecen, Böszörményi Street 138, 4032 Debrecen, Hungary; 4 Szent István Campus, Institute of Animal Sciences, Hungarian University of Agriculture and Life Sciences, Páter K. Street 1, 2103 Gödöllő, Hungary; fazekas.natasa@uni-mate.hu

**Keywords:** body dimensions, k-means clustering, Limousin, live weight, weaned calves

## Abstract

Despite the growing use of k-means clustering in cattle breeding research, its application to weaning performance traits in Limousin young bulls remains limited. This study evaluated the effectiveness of k-means clustering for identifying distinct groups based on age, live weight and morphometric records in a Hungarian Limousin nucleus herd. A total of 146 bulls weaned at an average age of 213 ± 2.03 days and live weight of 263 ± 3.31 kg (mean ± S.E.M.) were analysed. Age, live weight, withers height, rump height, back length, shoulder width, hip width and pin width were evaluated using k-means clustering. The optimal number of clusters was determined using the Elbow method, Average Silhouette Width analysis, the Calinski–Harabasz index, and the Davies–Bouldin index. Three clusters were identified, representing low (213 kg), intermediate (260 kg), and high (309 kg) body size groups. Live weight showed the strongest contribution to clustering (F = 387.3), followed by rump height (F = 85.6) and withers height (F = 82.8). Back length (F = 13.7) and pin width (F = 12.6) contributed the least. The greatest separation was observed between Cluster 1 and Cluster 3 (Euclidean distance = 99.26). Cluster 3 showed the highest within-cluster homogeneity, with no outliers and the lowest dispersion (SD = 10.67). These findings support data-driven phenotypic preselection strategies; however, future research should incorporate additional herds, economically relevant traits, and genomic data to enhance generalisability.

## Simple Summary

Identifying animals with superior growth potential at an early age is important for improving the efficiency of beef cattle breeding programs. K-means clustering is a statistical method that groups animals based on similarities in their traits. However, studies on its applicability in beef cattle growth and development have received limited attention. Therefore, this study aimed to investigate whether young bulls from a single Hungarian Limousin nucleus herd could be partitioned based on live weight and body measurements recorded at weaning. The results showed that animals could be successfully classified into lower, intermediate, and higher developmental levels. The group with the highest live weights and largest body dimensions showed the greatest uniformity among animals. Live weight, withers height, and rump height were the most relevant traits contributing to group differentiation. These findings demonstrate that routinely collected body measurement data are relevant from both statistical and biological perspectives and provide a foundational framework for data-driven selection and breeding strategies. To address current limitations, future research should be extended to more farms and additional economically relevant traits, while integrating precision technologies to enhance the generalisability of the conclusions.

## 1. Introduction

Clustering is a widely used method for identifying groups in multivariate data and is applied across fields such as biology, agriculture, economics, and machine learning [1,2]. It has been extensively used for tasks like classification, image segmentation, and data mining [3,4]. Clustering methods are generally divided into hierarchical methods, k-means (partitioning) methods and mixed approaches [5]. Among these, k-means is popular due to its simplicity and speed. It partitions data into k clusters, assigning each data point to the nearest centroid based on squared distances [2]. K-means clustering was originally designed for solving the single-view data clustering problem, but with the growth of large datasets, robust, multi-view k-means clustering methods have been developed to handle heterogeneous, more complex data [6].

K-means clustering is a core analytical tool in both beef and dairy cattle management, revealing intricate patterns in herd performance data. In dairy systems, this partitioning method is widely used to categorise cows by production, fertility, and health status (e.g., mastitis monitoring) [7,8,9,10]. Beyond traditional performance metrics, the algorithm is increasingly applied to genomic breeding values to support selection and enhance genetic progress [5,11,12]. In beef cattle research, k-means clustering reliably helps distinguish quality grades based on weight traits and body conformation, supporting decision-making in breeding and feed management [13,14,15]. Research indicates that clustering of ruminal microbial communities in beef cattle can aid in estimating feed efficiency and growth [16]. Recent studies have expanded its scope to secondary traits including reproduction and animal welfare and behaviour [17,18,19], as well as to the assessment of the economic efficiency of beef production [20]. Incorporating clustering data into AI systems enables predictions from real-time data inputs to optimise feeding strategies and improve health and productivity [21].

Despite the increasing use of k-means clustering in dairy and beef cattle research, limited information is available on its application to evaluating morphometric and weaning performance traits in Limousin young bulls. Therefore, this study aimed to investigate whether animals could be effectively grouped by live weight and morphometric parameters using k-means clustering analysis. This statistical approach may be a useful tool for identifying biologically meaningful subpopulations within the herd that share similar growth and body conformation characteristics. We hypothesised that morphometric and growth performance traits would exhibit sufficient variation to yield well-defined, biologically meaningful clusters of Limousin young bulls. Furthermore, we expected that the identified clusters would differ significantly in live weight and body dimensions, thereby facilitating the identification of phenotypically favourable groups of animals for preliminary selection purposes. These findings could establish a framework for developing preliminary selection methods using routine phenotypic data and support data-driven decisions in cattle breeding programs.

## 2. Materials and Methods

### 2.1. Ethical Statement

Ethical clearance was granted by the Institutional Animal Welfare and Use Committee of Széchenyi István University, Faculty of Agricultural and Food Sciences (Clearance Number: SZE-AKMK/MAB/012/2024).

### 2.2. Study Population

Altogether, 146 bulls born between January and September 2020 and between February and October 2021 were studied. Bulls were weaned at an average age of 213.38 ± 2.03 days (mean ± S.E.M.) with an average live weight of 263.00 ± 3.31 kg (mean ± S.E.M.). The farm operated a continuous calving system. The animals, based on the results of self-performance tests (between 210 and 450 days of age, according to the Association’s guidelines) and pedigree evaluation, were either marketed as breeding bulls or slaughtered (at around 400 to 420 days of age). Animals intended for slaughter were sold after weaning at a weight of 250–300 kg (max. 320 kg). The initial herd was imported from France, and has been further selected for key traits such as body frame, calving ease, soundness and rump width, which are important characteristics of breeding-type Limousin cattle. Imported and home-bred animals regularly replace breeding bulls. Performance data on weaning bulls indicate continuous genetic improvement in line with breeding objectives.

### 2.3. Husbandry and Feeding Conditions on the Farm

The study was conducted on one of the largest Limousin nucleus herds in Hungary, comprising 305 cows located in Nemesvámos, Veszprém County (47.0559° N, 17.8702° E). The mean temperature was 11.2 °C and total rainfall was 864.4 mm during the study period. Cows were managed in a pasture-based system, while weaned calves, heifers, and bulls entering self-performance tests were housed separately in deep-litter systems (heifers also had access to pasture). Calves remained with their dams until weaning and received starter feed in a calf-kindergarten.

The farm produced its own forage, consisting of grass and alfalfa hay. The grass species included in the hay mixture were white clover (*Trifolium repens*), tall fescue (*Festuca arundinacea*), perennial ryegrass (*Lolium perenne*), cat grass (*Dactylis glomerata*), alfalfa (*Medicago sativa*), Italian ryegrass (*Lolium multiflorum*), and meadow fescue (*Festuca pratensis*). In contrast, the granulated concentrate feed provided to all calves was a commercial formulation specifically developed for Limousin cattle. The concentrate was mixed on farm using an on-site feed mixer and contained barley, corn, wheat bran, pelletised malt sprouts, sunflower meal (36%), extracted soybean meal (46%), dried sugar beet pulp, limestone premix, sodium chloride, Immunowall, and magnesium oxide. The manufacturer provided the declared nutritional composition of this concentrate (Table 1). The micronutrient composition of the grass and alfalfa hay was analysed in the laboratory using a PerkinElmer Avio 550 MAX ICP-OES (PER-FORM Hungária Ltd., Budapest, Hungary) (except for selenium, which was analysed using a PerkinElmer NexIon 2000 ICP-MS (PER-FORM Hungária Ltd., Budapest, Hungary). The results are presented in Table 2.

### 2.4. Data Collection

**Figure 1:**
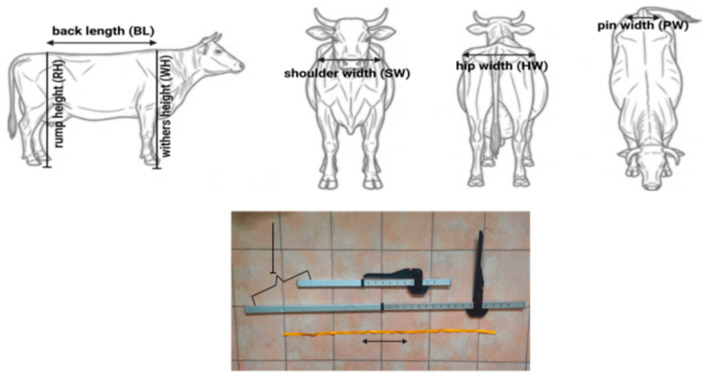
Anatomical reference points and tools used for body dimension measurements. Created in Biorender. Lili Dóra Brassó. (2026) https://BioRender.com/. Bars indicate measurements using a measuring stick, while arrows indicate tape measurements. Description of body dimension points: withers height—vertical distance between the ground and the withers; rump height—vertical distance between the ground and the highest point of the rump; back length—horizontal distance between the withers and the loin; shoulder width—horizontal distance between the left and right shoulder points measured across the withers; hip width—horizontal distance between the two hip bones; pin width—horizontal distance between the two ischial tuberosities.

Body dimensions were recorded in accordance with the breeding program of the Limousin and Blonde d’Aquitaine Breeders’ Association, which is mandatory for all participating farms. The aim was to obtain measurements that best represents the calves’ condition and development at weaning. Calves typically experience temporary weight loss due to weaning stress, whereas by the second week post-weaning, their physiological condition generally returns to a state close to pre-weaning. Therefore, data on body dimensions were recorded within two weeks post-weaning by an official delegate of the Association. The selected morphometric traits were chosen for their relevance to preselection and their usefulness as indicators of growth and body development. The same trained professional carried out all measurements to ensure consistency and reliability. The anatomical reference points used for body dimensions are illustrated in Figure 1.

### 2.5. Statistical Analysis

Statistical analyses were performed using SPSS (Statistical Package for Social Sciences) version 24.0 (IBM Corp., Armonk, NY, USA). Data normality was assessed using the Shapiro–Wilk test. In cases where the calculation did not confirm the normal distribution, we interpreted the data based on Q-Q and boxplots. The outliers were identified using the interquartile range (IQR) method, and observations falling 3 x IQR below Q1 or above Q3 were considered outliers. These values were removed before clustering to avoid their influence on cluster formation.

**Figure A1:**
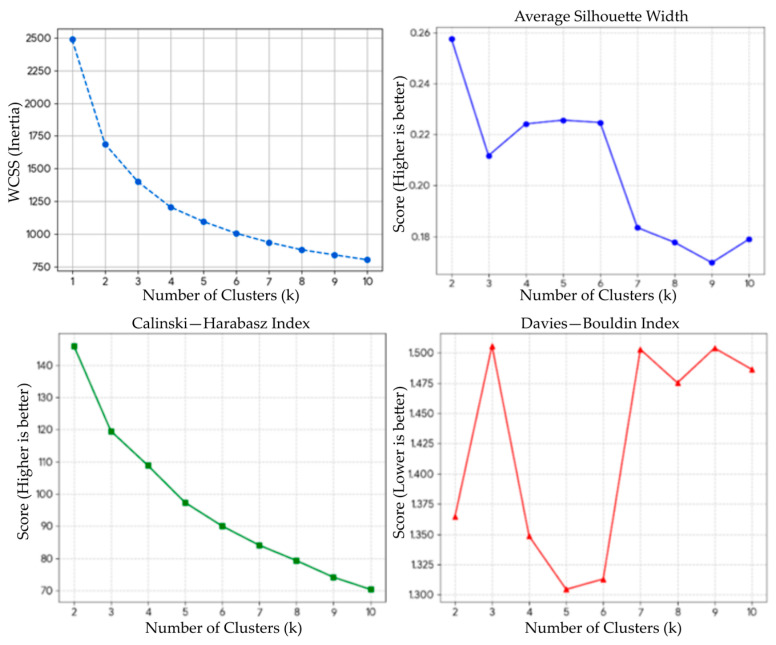
Elbow (WCSS), Average Silhouette Width, Calinski–Harabasz, and Davies–Bouldin methods used to determine the optimal number of clusters.

K-means clustering was applied to classify animals into homogeneous groups based on age, live weight and morphometric traits. The optimal number of clusters was determined using several validation methods, including the Elbow method based on the within-cluster sum of squares (WCSS), Average Silhouette Width analysis, the Calinski–Harabasz index, and the Davies–Bouldin index [22,23]. While the Average Silhouette Width and Calinski–Harabasz indices suggested a 2-cluster structure, the Davies–Bouldin index indicated an alternative solution of 4 or 5 clusters. These results demonstrated that different statistical criteria led to divergent optimal solutions: the 2-cluster solution lacked sufficient differentiation, whereas the 4- or 5-cluster models resulted in over-segmentation. In contrast, the WCSS values decreased substantially between *k* = 1 and *k* = 3, followed by a more gradual decline. When considered as a whole, these metrics indicated that the three-cluster solution provided the most appropriate balance between cluster compactness and model interpretability, both from a pragmatic and from an interpretation-driven perspective (Appendix A, Figure A1). The clustering procedure was based on the following variables: live weight (kg), morphometric measurements (withers height, rump height, back length, shoulder width, hip width, pin width; cm) and age (days). Euclidean distance was used as the similarity measure for cluster formation according to the following equation:

$$d_{\left(i,j\right)}=\sqrt{\sum_{k=1}^{p}{\left(X_{ik}-Y_{jk}\right)}^{2}}$$

where *d_ij_* represents the Euclidean distance between observations *i* and *j*, and *p* indicates the number of variables included in the analysis in a multidimensional data matrix, where *X* and *Y* indicate coordinates in a single dimension [24,25]. Cluster stability was assessed based on centroid convergence during iterative classification. One-way ANOVA F-tests were used to evaluate the contribution of each variable to cluster formation. Variables with higher F-values showed a greater contribution to cluster differentiation.

## 3. Results

### 3.1. Determination of Initial and Final Cluster Centroids

Initial cluster centroids were defined before the iterative classification of animals into homogeneous groups. These centroids were the starting reference points used by the k-means algorithm and served as temporary cluster centres, with animals assigned to the nearest centroid. Initial centroids reflected three size categories. Preliminary differences among clusters indicated a gradual increase in live weight and body dimensions from Cluster 1 to Cluster 3. Cluster 1 was characterised by animals with intermediate age (217 days) and the lowest live weights and body dimensions (live weight: 158 kg; WH: 92 cm; and RH: 99 cm), indicating smaller, less developed animals. Cluster 2 showed intermediate values for all traits (age: 166 days; live weight: 257 kg; WH: 107 cm; and RH: 117 cm), whereas Cluster 3 exhibited the oldest animals (248 days) with the highest live weights (live weight: 340 kg; WH: 116 cm; and RH: 126 cm) and the largest body dimensions, corresponding to larger and more developed animals.

**Figure 2:**
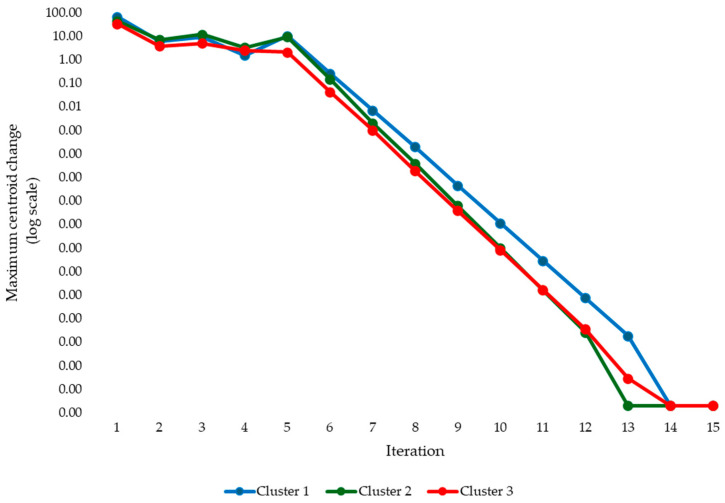
Convergence of the k-means algorithm across iterations (log_10_ scale).

The iteration history of the k-means clustering process presented in Figure 2 showed a progressive reduction in the maximum centroid change. The algorithm converged after 15 iterations, with only negligible changes in centroid positions. The final convergence criterion was 0.000, indicating that the k-means algorithm had successfully converged. Therefore, the final three-cluster structure could be considered stable and reliable, and it was suggested that further iterations would not substantially modify the classification of animals.

Final clustering was conducted to refine the results and obtain the final set of clusters for analysis. Final cluster centroids revealed a clear hierarchical structure among the three groups (Table 3). The descriptive characterisation of final clusters indicated that Cluster 1 consisted of calves with the lowest mean live weight and the smallest body dimensions, whereas Cluster 3 included the heaviest animals with the largest body dimensions. Cluster 2 showed intermediate values for most traits. The most pronounced differences were observed in live weight, withers height and rump height, since these traits contributed substantially to the hierarchical separation of clusters.

### 3.2. Cluster Separation and Validation

**Figure 3:**
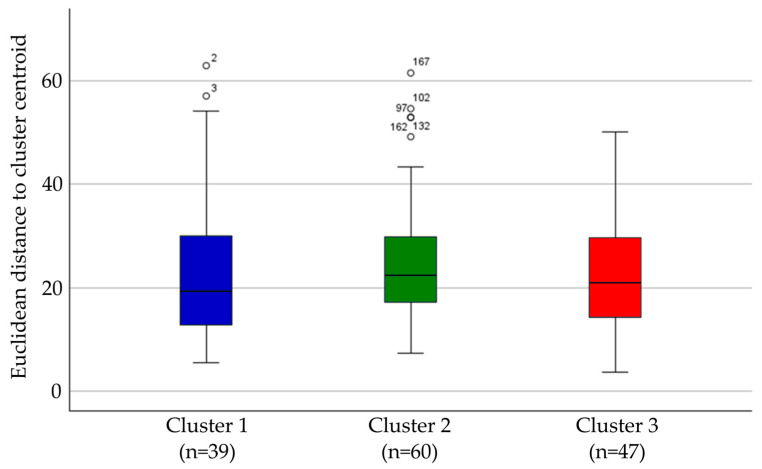
Distribution of individual Euclidean distances from their respective cluster centroids.

Figure 3 illustrates the distribution of Euclidean distances within clusters using boxplots, providing a visual assessment of cluster cohesion and homogeneity. The higher the Euclidean distance, ranging from 0 to 100, the greater the difference between two clusters. The inter-cluster Euclidean distance between Cluster 1 and Cluster 3 was the highest (99.26), indicating the greatest difference between these groups. Cluster 2 exhibited intermediate distances to both clusters, with values of 51.48 between Cluster 1 and Cluster 2, and 49.42 between Cluster 2 and Cluster 3, supporting its intermediate position within the cluster structure. Within-cluster homogeneity was assessed based on the interquartile range (IQR), the standard deviation (SD) of Euclidean distances to cluster centroids, and the number of outliers. These observed statistical indices showed that the mean cluster distance from the cluster centroid was 23.03 for Cluster 1, 24.73 for Cluster 2, and 22.50 for Cluster 3. The standard deviation (SDs) and IQRs for the clusters were as follows. SD = Cluster 1: 14.32, Cluster 2: 11.93, Cluster 3: 10.67; IQR = Cluster 1: 17.08, Cluster 2: 12.35, Cluster 3: 15.41. Cluster 3 had no outliers, while limited numbers of outliers were detected in Clusters 1 and 2. Based on the number of outliers and standard deviation, Cluster 3 showed a high degree of within-cluster homogeneity. However, Cluster 2 indicated the lowest IQR, suggesting a compact central distribution of observations.

### 3.3. Correlation Matrix Among Cluster Variables

Table 4 represents the correlations between the different variables, including age, live weight and morphometric traits. We could identify weak (r < 0.4) negative correlations between age, SW and PW, and between PW and BL. There were weak positive correlations between age, live weight, WH, RH, and HW. Age was weakly correlated with live weight, and PW was weakly correlated with WH and RH. BL was weakly positively correlated with live weight, WH, SW, and PW. Moderate (r = 0.4–0.7) positive correlations were identified between age and BL, and live weight and SW, HW, and PW. WH was moderately positively correlated with SW and HW. RH was moderately positively correlated with BL, SW, and HW. HW was moderately positively correlated with PW. PW was moderately positively correlated with SW and RH. Furthermore, live weight was strongly (r = 0.7–0.8) positively correlated with WH and RH, and SW had a strong positive correlation with HW. Very strong (r> 0.9) correlation was demonstrated between WH and RH. The results of correlation were statistically proven (*p*< 0.05 for BL-PW and *p*< 0.01 for all other variables), with the exceptions of age–SW (*p* = 0.397) and age–HW (*p* = 0.093) associations.

### 3.4. The Results of One-Way ANOVA Analysis

One-way ANOVA was performed to evaluate the contribution of each trait to the differentiation of the three clusters. The magnitude of the F-values showed that live weight, rump height, and withers height contributed most strongly to cluster separation, whereas back length and pin width contributed the least (Table 5). The variables did not contribute equally to cluster separation, as reflected by the magnitude of their F-values. Live weight showed the highest contribution to cluster differentiation (F = 387.3), indicating the greatest variations between groups, followed by rump height (F = 85.6) and withers height (F = 82.8). In contrast, back length (F = 13.7) and pin width (F = 12.6) contributed the least to cluster separation, as indicated by the lowest F-values. This implies that the clusters were most similar with respect to these traits and showed the least effective cluster differentiation. Shoulder width (F = 39.7) and hip width (F = 43.0) showed a moderate effect on cluster separation. Age differences among clusters were less pronounced than differences in live weight and body dimensions (F = 15.3).

## 4. Discussion

This study aimed to evaluate the applicability of k-means clustering to classify Limousin young bulls using live weight and morphometric traits. The identification of distinct morphometric clusters provided a practical basis for the early phenotypic classification of beef calves. It supported the development of cost-effective, data-driven preselection strategies based on routinely collected field data. The findings confirmed that k-means clustering could effectively distinguish biologically meaningful groups of calves based on live weight and body dimension data.

### 4.1. The Effectiveness of k-Means Clustering in the Classification of Limousin Young Bulls

K-means clustering revealed a clear separation between the identified groups based on live weight and morphometric traits, indicating substantial phenotypic variation within the studied population. The observed differences in within-cluster homogeneity and compactness further supported the robustness of the clustering structure, with Cluster 3 exhibiting the highest internal cohesion. These findings suggested that the applied clustering method could effectively separate biologically meaningful subpopulations using routinely collected phenotypic data.

Consistent with the present results, previous studies have also demonstrated the applicability of k-means clustering in the phenotypic classification of cattle and buffalo populations. Krupová et al. [9] identified three distinct clusters based on herd size, management characteristics, breeding values and production performance, reflecting different breeding system strategies relevant to cattle breeding programs. Similarly, Trapanese et al. [26] reported the presence of two or three distinct buffalo population groups depending on herd characteristics, using milk production and reproductive performance variables as clustering parameters. In dairy cattle, the k-means method has also been successfully applied to classify genomic breeding values based on milk production traits, identifying two major genomic groups [5]. Tőzsér et al. [27] evaluated the results of self-performance tests of candidate bulls from various beef breeds and could distinguish three clusters. Initial clustering revealed that the first and second clusters were closest after four iterations. Final cluster centroids indicated that animals in the first and second groups had higher weight gains and genomic scores. Furthermore, Wijebandara et al. [28] demonstrated the effectiveness of k-means clustering in differentiating dairy cattle populations by production and reproductive performance, including milk production, lactation length, and calving interval, and identified three clusters. In summary, these findings, together with the present results, indicate that k-means clustering is a reliable and biologically relevant multivariate approach for separating cattle subpopulations based on growth, production, reproductive, and morphometric traits, thereby supporting data-driven breeding and management strategies.

Although the clustering structure proved stable, the relatively small sample size may not have fully captured the phenotypic diversity of the Limousin breed.

### 4.2. The Importance of Live Weight and Morphometric Traits

In the present study, live weight had the strongest contribution to clustering. Multivariate clustering provided additional information beyond a simple ranking by weaning weight because cluster assignment was based on the combined variation in morphometric traits. Consequently, animals with similar body weights could differ in body conformation. This enabled the assessment of overall body development rather than live weight alone. Previous studies have revealed moderate to strong associations between live weight and body conformation traits in cattle populations [29,30,31,32,33]. Among the evaluated morphometric variables, withers height (WH) and rump height (RH) showed the greatest contribution to cluster separation, suggesting that these growth traits are important indicators of growth performance in Limousin young bulls. The substantial contribution of withers height and rump height to cluster differentiation is consistent with previously reported strong positive correlations between these body dimensions and live weight in cattle populations (r = 0.613–0.720 between WH and live weight; r = 0.593–0.750 between RH and live weight) [34,35,36]. These values were consistent with our results for the same traits. Furthermore, Yakubu [37], in White Fulani cows, also suggested a very strong correlation between RH and WH (r = 0.980), as well as live weight and body dimensions (r = 0.610–0.940). However, these author indicated moderate correlations between WH and SW (r = 0.510), similar to our findings. Slimene et al. [38] stated that these two body conformation traits are among the most relevant indicators for beef cattle grading. In Limousin cattle, Rezende et al. [39] reported a strong correlation (r = 0.760) between live weights measured at 210 and 365 days of age, indicating that early growth performance is a valuable predictor of later body weight and may therefore be used for early selection. Furthermore, Gunawan and Jakaria [40] found positive correlations between withers height and body weight at weaning (r = 0.328) and at one year of age (r = 0.782), supporting the use of body measurements as indicators of growth performance in cattle. Consequently, these morphometric traits likely reflect differences in physiological maturity and growth intensity in beef cattle.

The clustering procedure was based exclusively on the evaluation of phenotypic and morphometric traits measured in the post-weaning period. Despite their importance and usefulness in growth and development analysis, additional potentially influencing factors, such as genetics, maternal effects, health status and feed efficiency, were not incorporated into the study. Furthermore, the strong correlations between morphological traits (e.g., WH–RH r = 0.920; live weight–WH/RH r = 0.777–0.791) may have reduced their independent contributions to cluster formation. Raw weaning weight and age were retained to reflect practical on-farm conditions and enable farmers to apply these methods in their populations to group their animals based on clustering. Although there were relatively weak correlations between age and morphometric traits, we acknowledge that using unadjusted data meant that, in some cases, individuals with higher body weights were older bulls. Therefore, in future studies age-adjusted weaning weights (e.g., 205/210-day) should be incorporated when developing cluster centroids to more effectively separate the effects of live weight and morphometric traits.

### 4.3. Biological Interpretation of the Clusters

Cluster analysis evaluated traits based on intra-group similarity and inter-group dissimilarity [41]. The identified clusters in the present study likely reflected biologically meaningful differences in the growth performance and developmental status of young Limousin bulls. Animals assigned to Cluster 1 exhibited the lowest live weights and body dimensions, which were consistent with a less advanced growth status and body development at weaning. Conversely, Cluster 3 included calves with the highest live weights and largest body dimensions, suggesting enhanced body development and superior growth status at weaning [42]. The superior live weight and body dimensions of animals in Cluster 3 suggested accelerated skeletal growth and more advanced physiological maturity [27]. Cluster 2 represented an intermediate growth category. Furthermore, evaluating within-cluster homogeneity using Euclidean distances to cluster centroids showed that Cluster 3 exhibited the highest internal cohesion, indicating a more uniform and phenotypically consistent growth pattern among calves with superior morphometric development. Tőzsér et al. [42] assessed Limousin bulls of a mean age of 283 days and reported a live weight of 242 kg, WH of 105 cm, RH of 111 cm, BL of 68 cm, SW of 20 cm, HW of 32 cm, and PW of 11 cm. Although the animals examined by the authors were older than those in our three cluster groups, their morphometric measurements were generally lower, except for back length. These differences could be attributable to variations in husbandry practices, genetic background, feeding and environmental conditions. Furthermore, Tőzsér et al. [27] indicated that higher body weight was associated with greater body frame size. The first group was characterised by outstanding growth capacity and growth intensity but poor bone fineness. In contrast, the second and third groups exhibited the opposite pattern, showing lower growth capacity and growth intensity but greater bone fineness. Consistent with our findings, Slimene et al. [38] identified four distinct clusters of cattle based on morphometric traits, genotypes, and phenotypic characteristics. Two groups represented opposite phenotypic extremes: one was characterised by low live weight and smaller body dimensions, whereas the other showed higher live weight and larger body dimensions. One group was characterised by moderate live weight and superior body dimensions, while the other exhibited greater live weight but relatively moderate morphometric development. Since the animals in our study were maintained under similar feeding and husbandry conditions, the detected variation likely reflected individual differences in growth performance and developmental dynamics.

The studied population originated from a single-nucleus herd maintained under identical feeding, husbandry and management conditions. While this provided a controlled background for evaluating phenotypic variation among animals, it might have limited the generalisability of the results. So, caution is warranted when extrapolating these cluster structures to cattle populations raised under different production systems and environmental circumstances. Since calves were investigated at a single post-weaning stage, the identified clusters reflected developmental differences at that age and might not have persisted into later growth stages. Furthermore, the study did not account for maternal effects, including dam parity, age, milk production, and fertility, which may substantially influence calves’ growth performance and morphometric development. Therefore, future studies should incorporate data on dams’ age and maternal traits, such as milk production, fertility and parity, to better distinguish between calf- and dam-related sources of variation in growth performance and to strengthen the biological interpretation of the observed clustering patterns.

### 4.4. Practical Implications of Phenotypic Clustering

The identification of live weight, withers height and rump height as the major contributors to cluster differentiation might have practical relevance for the phenotypic assessment and preselection of young bulls. The continuous monitoring of cattle morphology and growth provided valuable information on animal development and welfare and served as an indicator of breeding efficiency [43]. Recent research on beef breeding systems has highlighted the importance of growth performance and morphometric traits in defining breeding objectives and improving production efficiency [9,44]. Furthermore, k-means clustering enabled the precise and objective identification of population structures that support the understanding of production and morphometric dynamics and data-driven decision-making by using routinely collected phenotypic records [26]. Moreover, the moderate-to-high heritability estimates reported for several morphometric traits (h^2^ = 0.25–0.68) [45,46] supported their use as essential selection indicators for improved growth and meat production performance. Therefore, the integration of routinely measured morphometric traits and clustering approaches might contribute to objective, cost-effective preselection and herd management strategies in beef cattle production systems. The identified cluster centroids may serve as a practical reference for classifying future calves originating from the same herd or a farm managed with identical genetic, climatic, husbandry and feeding conditions, without necessarily re-running the algorithm. However, when substantial differences exist in the mentioned circumstances, the original cluster centroids may no longer be representative. In such cases, the clustering procedure should be repeated with a new dataset to provide population-specific clustering and biologically meaningful data interpretation.

## 5. Conclusions

The study demonstrates that k-means clustering can be an effective tool for characterising phenotypic variation in Limousin young bulls. The identified clusters reveal meaningful differences in growth performance and body conformation, highlighting the potential of the clustering approach for the objective phenotypic classification of animals based on routinely collected performance data. The findings are relevant from statistical and biological perspectives and provide a framework for data-driven phenotypic preselection strategies. To address current limitations, future research should include animals from multiple herds and production systems as well as additional economically relevant traits. In the future, integrating pedigree information, estimated breeding values, genomic and longitudinal performance records, and precision technologies such as machine learning would help determine whether the identified clusters are associated with differences in genetic merit and long-term productive performance.

## Figures and Tables

**Table 1:** Nutritional composition of concentrate feed provided for calves on a DM (dry matter) basis (%).

Nutrient	Content on DM (Dry Matter) Basis (%)
Moisture	9.77
Crude protein	20.12
Crude oils and fats	2.71
Crude fibre	7.24
Crude ash	9.05
Ca	1.61
P	0.67
Mg	0.35
Na	0.37
Vitamin A (IU/kg)	15.80
Vitamin D_3_ (IU/kg)	3.16
Vitamin E (mg/kg)	52.64
Zn (mg/kg)	97.99
Cu (mg/kg)	21.01
Mn (mg/kg)	82.36

Data provided by the manufacturer on a dry matter basis.

**Table 2:** Mineral composition of grass and alfalfa hay (mg/kg of DM).

Compound	Content (mg/kg of DM)	
	Grass Hay	Alfalfa Hay
Ca	7020	5000
K	11,060	6300
Mg	1920	1240
Na	180	20
P	1160	1490
S	1190	920
Cu	5.31	3.09
Zn	16.57	18.3
Mn	74.17	43.6
Se	0.14	0.05

**Table 3:** Final cluster centroids including the number of animals in each cluster, total number of animals, mean, standard deviation (S.D.), and minimum and maximum for traits including age, live weight, and body dimensions.

Trait	Cluster	N	Mean ± S.D.	Minimum	Maximum
Age (days)	1	39	198.21 ± 19.43	166	250
	2	60	218.23 ± 22.87	166	280
	3	47	220.26 ± 17.21	183	263
	Total	146	213.53 ± 22.19	166	280
Live weight (kg)	1	39	213.08 ± 17.47	158	240
	2	60	259.73 ± 13.76	227	282
	3	47	308.59 ± 17.03	285	345
	Total	146	263.00 ± 39.97	158	345
Withers height (WH; cm)	1	39	103.21 ± 3.91	92	111
	2	60	107.98 ± 3.15	96	113
	3	47	112.21 ± 2.67	104	118
	Total	146	108.07 ± 4.72	92	118
Rump height (RH; cm)	1	39	111.77 ± 4.18	99	119
	2	60	117.00 ± 2.95	109	123
	3	47	121.26 ± 3.04	112	127
	Total	146	116.97 ± 4.93	99	127
Back length (BL; cm)	1	39	57.41 ± 4.39	46	67
	2	60	60.53 ± 4.48	53	70
	3	47	61.98 ± 3.17	56	72
	Total	146	60.16 ± 4.42	46	72
Shoulder width (SW; cm)	1	39	22.44 ± 2.08	18	26
	2	60	24.55 ± 2.16	20	32
	3	47	27.38 ± 3.37	22	36
	Total	146	24.90 ± 3.21	18	36
Hip width (HW; cm)	1	39	29.97 ± 2.35	25	35
	2	60	32.78 ± 2.37	26	39
	3	47	34.87 ± 2.61	30	41
	Total	146	32.71 ± 3.07	25	41
Pin width (PW; cm)	1	39	11.67 ± 1.18	9	14
	2	60	11.95 ± 1.14	9	15
	3	47	12.77 ± 0.89	11	14
	Total	146	12.14 ± 1.16	9	15

**Table 4:** Correlations between age, live weight and morphometric traits.

Variable	Age (Days)	Live Weight (kg)	Withers Height (WH; cm)	Rump Height (RH; cm)	Back Length (BL; cm)	Shoulder Width (SW; cm)	Hip Width (HW; cm)	Pin Width (PW; cm)
Age (days)	1.00	0.315 ***	0.262 ***	0.289 ***	0.553 ***	−0.022 ^ns^	0.110 ^ns^	−0.285 ***
Live weight (kg)	0.315 ***	1.00	0.777 ***	0.791 ***	0.382 ***	0.621 ***	0.582 ***	0.436 ***
Withers height (WH; cm)	0.262 ***	0.777 ***	1.00	0.920 ***	0.345 ***	0.529 ***	0.580 ***	0.398 ***
Rump height (RH; cm)	0.289 ***	0.791 ***	0.920 ***	1.00	0.412 ***	0.484 ***	0.583 ***	0.398 ***
Back length (BL; cm)	0.553 ***	0.382 ***	0.345 ***	0.412 ***	1.00	0.201 **	0.341 ***	−0.147 *
Shoulder width (SW; cm)	0.022 ^ns^	0.621 ***	0.529 ***	0.484 ***	0.201 **	1.00	0.744 ***	0.541 ***
Hip width (HW; cm)	0.110 ^ns^	0.582 ***	0.580 ***	0.583 ***	0.341 ***	0.744 ***	1.00	0.452 ***
Pin width (PW; cm)	0.285 ***	0.436 ***	0.398 ***	0.398 ***	−0.147 *	0.541 ***	0.452 ***	1.00

* denotes *p* ≤ 0.05; ** denotes *p* ≤ 0.01; *** denotes *p* ≤ 0.001; ns represents non-significance.

**Table 5:** One-way ANOVA results showing the contribution of each trait to cluster differentiation (mean squares, degrees of freedom, and F-values).

Variable	Cluster		Error		F-Value
	Mean Square	df	Mean Square	df	
Age (days)	6306.15	2	411.37	143	15.33
Live weight (kg)	97,776.09	2	252.46	143	387.29
Withers height (WH; cm)	865.05	2	10.44	143	82.84
Rump height (RH; cm)	959.02	2	11.20	143	85.61
Back length (BL; cm)	229.35	2	16.62	143	13.79
Shoulder width (SW; cm)	266.96	2	6.71	143	39.78
Hip width (HW; cm)	255.97	2	5.95	143	43.04
Pin width (PW; cm)	14.66	2	1.16	143	12.63

The contribution of each variable to cluster formation is indicated by its F-value, all of which are statistically significant (*p* = 0.001). This significance relates to the F-values themselves, rather than to the differences between the variables across the clusters. The F-tests should only be used for descriptive purposes, as the clusters have been chosen to maximise differences between cases in different clusters.

## Data Availability

The data analysed in this study were provided by the Association of Limousin and Blonde d’Aquitaine Breeders and are subject to third-party restrictions. Consequently, the data are not publicly available.

## Abbreviations

The following abbreviations are used in this manuscript:

DM	dry matter
WCSS	within-cluster sum of squares
LW	live weight
WH	withers height
RH	rump height
BL	back length
SW	shoulder width
HW	hip width
PW	pin width
df	degrees of freedom
